# Moving Toward Connectedness – A Qualitative Study of Recovery Processes for People With Borderline Personality Disorder

**DOI:** 10.3389/fpsyg.2019.00430

**Published:** 2019-02-28

**Authors:** Britt Kverme, Eli Natvik, Marius Veseth, Christian Moltu

**Affiliations:** ^1^Department of Psychiatry, District General Hospital of Førde, Førde, Norway; ^2^Department of Health and Caring Sciences, Western Norway University of Applied Sciences, Førde, Norway; ^3^Department of Clinical Psychology, University of Bergen, Bergen, Norway

**Keywords:** borderline personality disorder, recovery, treatment, qualitative, experiential

## Abstract

Borderline personality disorder (BPD) is a mental health disorder estimated to affect 1–2% of the general population. As a group, people with BPD endure a high degree of suffering, often leading to suicide attempts, self-harm, and suicide. Comparatively few studies explore the first person perspective of the person suffering from any mental health disorder. This might be especially problematic for people diagnosed with BPD, as this particular diagnosis is followed by stigma potentially making help seeking harder and helping relationships more vulnerable. We interviewed 12 female participants recently diagnosed with BPD in-depth about their experiences with recovery and treatment, and used a stepwise reflective approach to rigorously analyze the data. Results show an overarching theme of working toward connectedness, with four constituent sub-themes. We discuss the findings with regard to empirical work, recovery and autonomy, and the risk of epistemic injustice that people with BPD risk facing.

## Introduction

Borderline personality disorder (BPD) is a mental health disorder estimated to affect 1–2% of the general population (Swartz et al., 1990; Torgersen et al., 2001; Lieb et al., 2004). BPD is diagnostically characterized by identity disturbance, difficulties regulating emotions, impulsivity, and self-harm (Lieb et al., 2004). It has been suggested that persons diagnosed with BPD have difficulties with mentalization, and that BPD is best understood as a trauma- and attachment based disorder (Bateman and Fonagy, 2003). Suicide attempts are common among persons diagnosed with BPD. In a recent large scale survey the adjusted risk for having a recent suicide attempt with the presence of a BPD diagnosis was reported to be 13,55% (AOR, 13.55; 95% CI, 10.29–17.85) (Olfson et al., 2017), among the highest between all mental health disorders. Earlier research has demonstrated that between 3,0 and 9,5% end up taking their own life (Stone, 1989). The startling severity of suffering and risk associated with a BPD diagnosis underscore the importance of developing sound knowledge about the course of suffering and recovery for these people.

Developing effective treatments for people with BPD has been on the agenda during the past 20 years and specialized treatment programs, such as dialectical behavior therapy (DBT) and mentalization based treatment (MBT), have been developed (Linehan, 1993; Bateman and Fonagy, 2003; Kellogg and Young, 2006). Multiple RCT studies have demonstrated that these programs are effective in reducing self-harm, hospitalization, and suicide attempts incidents (Bateman and Fogany, 1999, 2008; Linehan et al., 1999, 2006). There is little evidence that medication for people with BPD holds positive treatment effects (Stoffers and Lieb, 2015). In conclusion, research has shown that the symptoms of BPD are treatable, primarily by psychological, social, and relational approaches rather than medication. However, less effort has been put into researching how the persons who are suffering and treated by some treatment modality experience these interventions and potential change processes.

In general, comparatively few studies explore the first person perspective of the person suffering from a mental health disorder (Flanagan et al., 2007, 2010), and/or being in treatment programs. The central focus is often on the intervention itself, and its effect toward some researcher-defined objectified outcomes on a symptom level. For general mental health suffering, multiple qualitative studies, and meta-syntheses have reported that important aspects beyond symptom reduction are salient to recovery. These include engaging in meaningful interpersonal processes, gaining understanding about one’s own patterns of suffering, finding hope and motivation to drive autonomous change, and taking responsibility for one’s own life (Young and Ensing, 1999; Veseth et al., 2012; Bjornestad et al., 2017; Hansen et al., 2017; Moltu et al., 2017; Lavik et al., 2018).

In the recovery tradition, the process of getting better is seen as a personal and continuous journey that revolves around learning to live with the challenges set by mental illness and restoring meaning in one’s social and personal life (Anthony, 1993). From this point of view, a treatment intervention is but one out of many situations and practices where the individual person works toward his or her recovery, with the potential of being of high and low importance to any specific individual. When focusing the research perspective on a specific professional intervention, studies risk losing sight of how any intervention is integrated as part into the whole of the suffering person’s life. Centring the research perspective on the suffering person’s experiences with moving through different situations in the process of working toward recovery holds the potential of a sound contextual understanding of treatment processes and their place within a person’s lifeworld. The lifeworld is our experiential everyday life, projects, social encounters and surrounding objects – a familiar and taken for granted world, and yet impossible to fully understand (Husserl, 1954/1970).

Developing detailed insight into how suffering is experienced for persons diagnosed with BPD, and understanding what are important arenas that recovery takes place in, are important areas of professional knowledge. Flanagan et al. (2007) underscore how persons’ lived experiences can contrast how symptoms and suffering are understood in the diagnostic literature. They exemplify with Miller’s (1994) qualitative study of people with BPD showing that the diagnostic criterion “markedly unstable sense of self” (American Psychiatric Association [APA], 2000) attains a very different meaning when studied from the first person perspective. Miller (1994) summarized that rather than having an unstable sense of self the participants in her study seemed to have a stable sense of being an impaired person, leading Flanagan et al. (2010) to argue that subjective data needs to be integrated in diagnostic systems. To this end, Black et al. (2014) have contributed a phenomenological study of the first person experiences of nine persons diagnosed with BPD. They describe tensions between working to stay present to experiences and disappearing for oneself through dissociation and suicidal fantasies. One important work to integrate subjective experience into the practices of assessment and diagnosis is the recently published psychodynamic diagnostic manual (Lingiardi and McWilliams, 2017), which includes sections on subjective experiences for each of the main chapters. The authors emphasize in their motivation for undertaking this work that “*Without a counterpoint to the current tendency to focus more and more narrowly on discrete disorder categories, the clinical relationship may be jeopardized and even damaged*” (Lingiardi and McWilliams, 2015).

Knowledge and understanding of recovery for people with BPD can support health professionals in their effort to support the patient in finding his or her own personal way of recovering from their illness, while at the same time as assisting the person by helping alleviating clinical symptoms (Davidson et al., 2005). Consequently, a need for knowledge from persons who suffer from BPDs’ first-person perspective and of how they experience their recovery in the context of treatment is evident. To contribute into these knowledge areas, we have chosen to explore the two research questions: How do people diagnosed with BPD experience processes of getting better? How do people diagnosed with BPD experience treatment interventions in their work to get better from suffering?

## Materials and Methods

### Participants

Eligible participants had had a diagnosis of BPD established between six and 18 months before inclusion. This criterion was used to invite people with relatively fresh experiences with having been given the diagnosis of BPD. In addition, eligible participants had to be currently in contact with a treatment provider. This project aimed to study people in ordinary settings, and did not carry out diagnostic assessments as part of procedures. In Norway, clinical psychologists and psychiatrists working in specialized mental health care settings are mandated to diagnose personality disorders in accordance with best practice. Normally, this is done within a clinical relationship based on clinical interviews and structured diagnostic interviews. Participants had gone through this process 6–18 months prior to participation.

Our exclusion criterion was active psychosis. Clinicians at four different hospitals were informed about the study and the inclusion criteria, and provided an invitational letter, explaining the scope of the study, to eligible patients. Eighteen women were invited; twelve women between 21 and 36 years from different parts of Norway agreed to take part in the study. The protocol did not define women as the target population, but diagnostic practice in the clinics led to only women meeting inclusion criteria. Table 1 presents a summary of the participants and the treatment contexts.

**Table 1:** Descriptive sample overview.

*N*	Gender	Ages	Treatment context
7	K	21–37	Current DBT context
1	K	26	Finished DBT, now TAU
1	K	36	Starting phase DBT
2	K	22–25	Current MBT context
1	K	27	Current MBT inspired TAU


*DBT, dialectical behavior therapy; MBT, mentalization based treatment; TAU, treatment-as-usual.*

### Researchers

BK is a clinical psychologist working in the public healthcare system. EN is an associate professor of health science. MV is an associate professor of clinical psychology. CM is a clinical psychologist working in the public healthcare system, and a professor of clinical psychology. The researcher group share an interest in humanistic health perspectives, service user participation in research, and qualitative health research. None of the researchers had had any treatment relationship with any of the participants in the study.

### Data Collection

To achieve the objective of studying the first person experiences of participants (Binder et al., 2012), individual in-depth interviews were chosen as the data collection method. This strategy is particularly apt when the aim is to explore personal experiences in-depth, and building safety for rich descriptions (Knox and Burkard, 2009; Kvale and Brinkmann, 2009). The group of researchers jointly developed an interview guide with broad questions directed at areas of interest, and strategies to move the conversation to the experiential level of each participant. BK traveled to the treatment location to which the participants were connected, and met with each participant in a designated interview room. Care was taken to create a safe and welcoming atmosphere and avoiding disturbances in the interviews. We conducted twelve interviews with twelve individuals, lasting from 45 to 150 min. All interviews were audio recorded and transcribed verbatim for analysis.

### Data Analysis

We used a systematic, team-based and step-wise reflective approach to rigorously analyze the participants’ experiences (Binder et al., 2012; Hill, 2012). The structure of the analytic process was Thematic Analysis aiming to establish emergent themes across the individual accounts (Braun and Clarke, 2006). We proceeded as follows. (1) All authors read the full material closely to get a basic sense and overview of the data material, and made preliminary process notes. (2) BK, EN, and CM met for a half-day analytic seminar to reflect on the data, look for patterns of meaning and organize focus for further analyses. (3) BK used the notes from the seminar and carried out a structured thematic analysis (Braun and Clarke, 2006), resulting in a group of preliminary themes with reference to different parts of the data. (4) BK, EN, and CM met for a full-day analytic seminar to work through and review preliminary themes. Adjustments to preliminary thematic structure were carried out. (5) BK refined the analysis, and considered the resulting thematic structure back with the interviews to ensure that meanings corresponded. (6) MV, who had not been part of the initial phases of analysis, reviewed the resulting themes in light of the data material, performing the function of a critical auditor (Hill, 2012).

### Ethical Considerations

Talking to persons diagnosed with BPD about mental health, relationships and suffering can be a very sensitive matter, demanding ethical reflection, and thoughtfulness. Choosing to invite people in current treatment contexts was one measure to ensure that participants had some level of support around them. Moreover, one of the researchers in the group, with longstanding clinical experience, were available to the more inexperienced interviewer should something come up in the interviews that caused concern for the participants’ well-being. Participation was explicitly voluntary, with anonymity guaranteed, and participants who volunteered gave written consent and were informed that they could withdraw this consent without consequences at any time up until the publication of an article. The Regional Ethics Committee for Medical Health Research approved of the study (REK Vest 2015/882), and the data security agent at Helse Førde approved of the data security protocol.

## Findings

We present the findings pertaining to how participants experience and describe recovery and its relationship to treatment through one overarching theme, “Moving toward connectedness.” Moving toward connectedness is a description of the most common theme across the participants’ experiences of treatment and recovery, and reflects the most abstracted level of our analyses. As an abstraction, it provides a structure for understanding the constituent subordinate themes. Four subordinate themes, “Learning to hold one’s own,” “Needing honesty and genuine mutuality,” “Daring to belong,” and “Making a room for recovery” describe important nuances and variations in the participants’ treatment-experiences as part of their recovery journey. The subordinate themes both contribute details to and are part of the overarching theme of connectedness.

### Moving Toward Connectedness

Connectedness was a central theme emerging in persons with BPD’s experiences with change and development during treatment. Connectedness expresses a sense of belonging to one’s own being in the world, and means holding oneself, holding on to oneself, taking a place in the world, and feeling part of it. Across participants, connectedness implied feeling “I am like others and others are like me,” feeling human amongst other humans. Being connected seemed to help the participants standing steadier in raging emotional storms, reaching for and clinging on to support from friends and family and to the support found within themselves. Although struggling and feeling swept away by strong emotions intertwined with processes of treatment and change, participants across different treatment contexts and at different stages in their processes shared the experience of moving toward connectedness.

### Learning to Hold One’s Own

To be agents in the processes of change was pivotal. Agency implied being able to believe that change could come about by acting, by changing old patterns and habits. In the context of treatment, this seemed sparked by the experience of being seen as one “who could” by one’s therapist, and by daring to embrace the faith that the therapist had in them. Some participants had started feeling that “they could,” and were actively engaged in helping themselves to make moment-to-moment decisions that could help them deal with their emotions without needing to self-harm, moving them forward in their personal recovery. Importantly, other participants struggled and did not currently experience the same degree of agency, or that others did not encourage agency to grow. They easily felt lost in destructiveness when it came over them, a state of darkened thought, emotions and self-abuse. Possibilities of wanted change, which had emerged to the participants who believed that they could, were illusive and hidden from the participants who believed “they could not,” as illustrated by the following quote.

When I’m home what am I supposed to fill the time with, that I have spent on other things? The whole of last semester I was drinking wine every day, one bottle of wine every day! What am I going to fill that time with that I used to drink? I stayed sober for almost 6 months, I’ve started to drink again now, but that’s like…what else am I supposed to fill the time with that I used to spend on drinking?

Even though this participant was in an extensive therapy program, she seemed to be unaware of the possibility that she could choose differently, that she held the power to make changes in her life. The participants who believed that they could, experienced having therapists who conveyed possibilities for how to make changes, and, as such, the therapists were carriers of hope. They were cheerleaders who made the participants feel significant and brave. A participant described that she had lived in a cave of blackness, of self-hatred and emotional pain. By being supportive and encouraging her therapist had made a profound difference in her life, helping her slowly to believe in herself again, daring her to step out of her blackened cave.

I don’t believe in myself, I have zero faith, but she believes. Oh my GOD she believes! It’s like “calm down, I’m not THAT good!” and she’s like “You can do it!” And that’s just crazy, to have someone saying your words, the words you should be saying to yourself and who will not be quiet about it. She will go on shouting “You can do this! Don’t give up!” That means everything!

This participant further expressed that her therapist’s engagement had made her take a hard look at her own way of living, facing her self-destructive eating and sleeping patterns, helping her to make small changes toward taking better care of herself. Some participants did not express that they were encouraged to help themselves. They were trying to *be* changed by others, having change induced *in* them, waiting for recovery to come *to* them. There where no echoes of cheers in these narratives. The quote below gives voice to the sound of powerlessness that was evident in the narratives of those who struggled to experience that they could.

I don’t know… I really don’t know how I am going to get better, I have enough with holding on to one day at a time.

The participants who believed they could, experienced that they had learned something through therapy; they had gathered knowledge about their emotions, their thoughts, their behavior, and how thoughts influenced emotions and behavior and vice versa. They had gathered keys they could use to unlock doors opening up various possibilities of change. Importantly, these keys were about doing; making conscious choices, being curious about their inner world and practicing how to use their keys. By having and using their knowledge and capacity to act, by taking a walk or cuddle up by the TV with a comedy, the participants experienced that they could turn away from destructiveness, altering the thoughts and emotions accompanying this darkened state. The following quote illustrated how one participant actively used her knowledge to break the emotional state she was in, finding a way to regulate her emotions without self-harming.

It’s about stepping out of the bubble you are in. When it explodes I am usually at home or somewhere else, so it is about doing something totally different. When you’re having a difficult time you often become inactive and just lay down you know, but if you go outside and do something, you trick your brain to say “Okay let’s do something constructive, let’s do something good.” So I usually go for a walk, I often start out very aggressive, hard pace, but eventually I calm down and when I get back I am usually fine.

One participant described how she felt herself growing stronger by witnessing her own journey from powerlessness to emergent control. Experiencing herself as one “who could” and seeing the changes she was making in her own life gave rise to feelings of pride, achievement and mastery.

P: I can make better choices now. So that’s the strength I feel I have regained. I’m not my old self, but now I’m on the right path, and I have NEVER felt that way before, at least not in the last 10 years (….) I have grown a lot by being here.”

I: sounds like a big change.

P: Absolutely! It might not be for you, but for me, even sitting here right now, I just want to scream out in joy! This is a feeling of achievement for me!

The process of change seemed to be about learning to hold one’s own, as described in the quote below. By using the analogy of building a house this participant explained how she, with the help of her therapist, was slowly rebuilding herself, learning how to manage standing on her own two feet.

(…) It’s like building a house, first you need stability in your daily life, work and somewhere to live, that becomes that steady foundation the house can be built on. Then you need a ladder, I’m on that ladder and the psychologist is giving me the tools so I don’t have to go up and down on that ladder, getting the tools, cause then I go down emotionally, if you think about it that way. Then I can be up there, growing, building and when the walls are up, and the roof is up that’s the year that have passed in treatment. Then the house is finished, but it’s never actually finished, I have to continuously work on it you know (..) but then it’s not such a big job that I need that ladder, then I don’t need the carpenters showing me how to use the tools, then I know how to use them myself.

The participants highlighted that being encouraged to learn how to help themselves interconnected with taking ownership of their own recovery. Experiencing ownership seemed to help the participants in the context of treatment, as well as empowering them to hold their own even after finishing their treatment programs.

### Needing Honesty and Genuine Mutuality

To feel validated and met as unique persons facilitated trust and security in the therapeutic relationship. Feeling seen as individual beings with emotions, thoughts and intentions, they experienced their therapist as someone who saw past psychiatry and took behold of the person behind. Many of the participants had experienced themselves as illusive, and had been negligent to their inner self. Through the experience of feeling understood, and by having their thoughts and feelings acknowledged and given meaning to, the participants seemed to become visible for themselves. They expressed an experience of finding “me” through the eyes of another that enabled them to respect, take care of and to care about themselves in a newfound way. Not every participant experienced feeling understood in the context of treatment. One participant who struggled, explained that she felt that her thoughts and emotions were perceived through her diagnosis, her own intentions second-guessed. In the quote below she described having to battle with her therapist to feel heard and understood.

I have told my therapist, and I feel that she doesn’t hear what I’m saying. That’s been really hard, cause then I feel that she makes it into having something to do with my illness. She will say “Yes, but a lot of people have difficulty with that, and relational issues and stuff.” But then I feel like she doesn’t even see me! My biggest problem is with myself and not other people!

The participant’s relationship to their therapists seemed very important for their recovery at the time we interviewed them. Therapists who made the participant feel understood and validated, who did not avoid uncomfortable issues, who did not hide away hurtful truths, and who were forthcoming and open about every turn in the process of recovery, were experienced as trustworthy, and genuine. Being able to reach the therapist outside of therapy was experienced as a form of scaffolding. Knowing that someone was available to offer support seemed to give the participants courage to stand on their own two feet. It could also wash away misunderstandings in the therapy room as illustrated it the quote below. This participant expressed feeling upset after a therapy session when her therapist had confronted her with her “habit” of pushing people away. Being able to send her therapist a text helped her to hold the feeling of rejection and to come out of the emotional state she was in.

I feel like she sees me, even if it really hurts. When I left I felt like a really bad person, I’m just bad at being a person I thought, I can’t accomplish anything. And then I sent my therapist a text. You know it’s just that, with my old therapist I couldn’t reach him by mail, text, nothing. There was no way of communicating with him until the next session. (…) So I was really upset and sent her a message on the way home, and I got this answer that really calmed me down. So then I felt like maybe it wasn’t so bad. She said that “Yes we all get blown off, it is not just you, we all get rejected” (..) That makes it more human you know, so then I felt I could relax and go on living.

Feeling safe and secure in the context of treatment enabled the participants to lay down their guard, focusing their attention on how to get better instead of standing watch, protecting themselves from harm. A participant, who struggled in her recovery, expressed that she experienced the absence of openness, mutuality, transparency, and trust in the relationship with her therapist. In the quote below she described how the distance between them seemed to expand by the titles of “therapist” and “patient.” The former holding the cards of treatment so the latter could not see them, creating insecurities and mistrust in the therapeutic relationship, seemingly hindering change instead of facilitating it.

I feel that it’s all on their terms. I know that the relationship to the therapist is very important, a lot of studies point to how important it actually is (..) and this is not a good relationship. My therapist has even told me that she might have to try to find me another program. And when I asked where she was going to send me she just “I don’t know, you might be too ill to be here” And I just, “Well isn’t this the kind of problems you should be able to handle?” So it’s been…very strange, either you are too ill or you are too healthy, it’s difficult to be a patient.

### Daring to Belong

Opening up to others and taking a genuine part in relationships in the treatment context were seen as important for recovery and difficult to approach. Group therapy sessions were potentially meaningful spaces, as a sanctuary where the participants’ own thoughts and feelings could resonate with those of others. The other people who were patients rather than the group therapist seemed to give structure to these experiences. One participant described how the group offered her a homelike feeling contrasting the feeling of separateness she often experienced in the outside world.

Group therapy probably helped me the most, cause then I met people who struggled with the same… as I felt I was the only one, in the whole world, who struggled with. Then suddenly I meet people with exactly the same thoughts and emotions as me. So that probably helped the most.

One participant disclosed that she felt a lot of shame over her illness. Within the group she experienced that she could talk about her life without being embarrassed or judged, giving her a feeling of being understood and accepted. Within the group, she was part of a community, part of a whole.

It was scary at first, but now I notice that I need these Thursdays! This is the only place where I can feel calm; nothing is embarrassing here, nothing stupid, and nothing to feel ashamed of! Here I get the feeling that people actually believe me, that I don’t get in my daily life surrounded by family and friends. I look well on the outside you know, but I feel ill inside. People can judge me and be like “but you’re not ill, you look so fresh and nice and have a tan.” But on the inside I look like a storm, you know!

To belong to a community gave rise to a feeling of togetherness. Togetherness was about learning and acting together, to help, and to encourage each other. Participants created it intentionally, supported by a structure within the treatment, bestowing continuity on a process of change.

However, when the architecture and elements of togetherness were absent, being in a group could be a lonesome experience for some, causing anxiety and feelings of shame. Being in a group meant talking about their daily life, and to face fellow group members’ opinions and views on one’s own thoughts and actions. For one participant, being in a group was not about feeling connected and whole; it was about feeling vulnerable and insecure, as illustrated in the quote below.

The group therapy has been really difficult. I struggled a lot with anxiety, taking up space in a group and starting to talk and to have all that attention pointed toward me. It has been really uncomfortable (..)

### Making a Room for Recovery

Recovery processes were connected to hope. Participants who experienced moving forward in the process of recovery expressed that they believed in possibilities of change, and were actively pursuing each step toward it. It seemed the participants had established a room in mind were they could see their own life as it previously had been, how it was now and how they wanted it to become. Having room in mind was also about being compassionate toward one self and the process one was in. The quote below illustrates how one participant reminded herself that she was “allowed to make mistakes” in the process of recovery.

To be satisfied with the little steps I take, or to me they are really big steps but to others they might be: “Really is that a problem?” I just have to be proud of what I accomplish. I have to try, and if I fail, well that’s okay too.

Some of the participants seemed to have created a timeline in their mind were each step in treatment signified a move forward toward better days. In the face of setbacks this timeline could re-establish hope, showing the progress that had been made and could be made again. Forms and notebooks helped the participants to visualize their timelines, as they could go back in these books and see their own progress, how it had unfolded over time. The quote below illustrated how one participant was able to reflect on her own progress, even in the face of setbacks, acting understanding toward herself, complimenting herself for the changes she had been able to make.

I had a setback this weekend (...) But even if I have these major setbacks once in a while they are shorter in duration. I can intervene in my own thoughts and think more rationally about what’s going on in my head, and that’s HUGE! To be able to step into your own line of thoughts, pull them apart and actually be strong enough to face the one’s you have acted out against and say “that wasn’t right of me to do.”

Making a room for recovery was about having patience with one’s own recovery process, considering one’s own timeline gently, and with care and compassion. For some this meant letting time pass by, letting the processes of change mature at its own pace. For one participant room was about feeling free to be skeptical and negative about her treatment for as long as it took for her to experience change.

P: (…) I was in treatment for one and a half year, you’re only supposed to have 1 year but I was able to have an extra 6 months. You go through the book once every 6 months, you know. The first 6 months I was really negative.

I: To the treatment?

B: Yes I thought it was ridiculous! But the next semester was better and the last semester was great!

Other participants did not experience being helped in making room to recover. One participant disclosed that she felt change was time-restricted and for a lucky few to experience. She described a feeling of being deserted, when being told that she had to end her treatment before feeling ready to do so, not knowing where to go or who to turn to, feeling guilty over not getting better in the time she was expected to.

(...) It had to have been some kind of miscommunication (..) cause when I had 1 year left my therapist came and told me that we had to talk because I was overstaying my invitation, so to speak. “You have to quit by Christmas” she told me. That was really devastating for me (...) I knew I had to quit and I was prepared, but in a year as they previously had told me. I totally lost it, it was just dreadful.It was such a mismatch between what I thought and what was.

Room to recover was something more than just time, as illustrated above, it was being granted humanity and individuality by others in a vulnerable period of one’s life, and by the individual herself making a compassionate room in mind to be allowed to recover. In the context of treatment, it seemed to present itself where it was made space for by therapists, by the means of flexibility, care and empathy.

## Discussion

In the findings of the current study, we have presented four themes that demonstrate variations and concretizations of the shared overarching theme of recovery for people diagnosed with BPD as a process of moving toward a personal sense of connectedness. Although the experience of becoming safer as a human among other humans constituted a core meaning of recovery, different participants found themselves on different paths of this journey. The four sub-themes, “learning to hold one’s own,” “needing honesty and genuine mutuality,” “daring to belong,” and “making a room for recovery,” address important ways that participants experienced that therapy could help or hinder their personal recovery processes. For example, some participants had experienced honest mutuality in their treatment relationships, and others described they were left longing for it. Between the two poles was a shared understanding that this was necessary to be able to use therapy for the good in the context of one’s own life. The sub-themes illustrate that for therapy to be of value, both relational aspects, such as honest mutuality and being invited to belong in groups, and support of autonomous aspects, such as holding one’s own and making room, interplay.

Qualitative explorations from a first-person- and therapist perspective describe the importance of belonging and social connectedness for people with severe mental illnesses (Bradshaw et al., 2007; Veseth et al., 2017; Ware et al., 2007). Bottom-up analyses of the literature (Drake and Whitley, 2014) and syntheses of existing qualitative studies (Leamy et al., 2011) emphasize belonging as decisive factors to processes of recovery, both through connecting to ones close relations such as family and friends, to professionals, and to the community and society at large. Performing a systematic review and meta-synthesis of qualitative studies of recovery for people diagnosed with personality disorders in general, that is, a much broader inclusion scope than people diagnosed with BPD, Shepherd et al. (2016) found only three studies to include. They report that the higher order themes of safety, social relationships and autonomy are understood as a prerequisite for an individual to recover. The latter part speaks to the core findings of the present study, with its focus on an individual being autonomous, but inextricably connected to a relational context. As such, our study adds to this growing knowledge base in highlighting the central role of connectedness to people with BPD.

Developments in psychotherapy research and theory for people with BPD have evolved over the past three decades, for example through mentalization-based treatment (MBT) (Allen and Fonagy, 2006; Bateman and Fonagy, 2013) and DBT (Linehan et al., 2001). Both MBT and DBT have proved effective in helping this group of people (Bateman and Fonagy, 2009; Kliem et al., 2010). MBT is an attachment based therapy form, in which BPD is both theorized (Allen and Fonagy, 2006) and empirically shown (Gullestad and Wilberg, 2011) to be associated with a lessened ability to “hold other minds in mind” based on (object)-relational trauma (Allen and Fonagy, 2006). A therapeutic focus in MBT therapies is to explore and approach relational situations while working to support emotion regulation and mentalization in processes of intrapersonal and relational healing toward greater epistemic trust (Fonagy and Allison, 2014). A third-wave cognitive therapy form, DBT integrates self-regulation, mindfulness, and acceptance in parallel individual and group settings (Linehan et al., 2001). Clinical recovery in DBT is directed at helping people with BPD with self- and interpersonal safety and regulation for participation in social contexts. As such, both these therapeutic traditions mirror the subjective experiences of the present study’s participants’ struggle toward connectedness, for example through MBTs focus on epistemic trust (Fonagy and Allison, 2014).

We may argue that processes of connectedness appear particularly important for people with BPD. Traditionally, a BPD diagnostic label has corresponded to that of a “difficult patient” (Sulzer, 2015) to people that in the mental health system often has been referred to as “manipulative,” “unstable,” “disinhibited” or “hostile” (Kyratsous and Sanati, 2017), leading helping systems to risk denying people with BPD the invitation to a genuine community to a larger extent than those with other ways of suffering. An experimental study by Lam et al. (2016) demonstrates the stigmatizing effect a BPD label can have on therapists’ assessment of uncomplicated panic disorder. Prior to watching a film of a woman describing her lived experiences of panic, the researchers randomly allocated therapists to one of three conditions. The first group was provided written information about the patient’s personal details and general background; the second had the addition of a behavioral description consistent with BPD; and the third group was given the addition of a label of BPD. The group provided with the label saw significantly fewer reasons to be optimistic about the woman’s prognosis and rated her problems more negatively. Similar to people with addiction problems risking rejection from helping professions when showing core feature of their problems, such as active use of illegal drugs, people with BPD can be seen to risk rejection for showing core features of theirs, such as reactions to relational trauma and insecurity. However, in light of now available psychotherapies helpful to people with BPD, such as DBT and MBT, there is also a flip side to these issues. Historically, limited access to care has been reported in studies of persons’ with BPD experiences (Nehls, 1999), and without a diagnosis, access to potentially helpful programs might continue to be limited.

Recently, adverse processes have been helpfully discussed using the concept of “epistemic injustice” in understanding how people with BPD are being met by helping professions (Kyratsous and Sanati, 2017). Diagnoses in the field of mental health are by different accounts claimed to be uncertain and fundamentally problematic (Bentall, 2006; Horn et al., 2007; Pilgrim, 2013), underscoring that they do not identify patients with common symptoms and etiologies who respond to specific treatments. However, as Pilgrim (2013) writes: “although psychiatric knowledge is weak, psychiatric authority is powerful” (p. 339). In a brief popular article problematizing the potential detrimental effects of this authority for people with BPD, Watts (2017) builds on the concept of “epistemic injustice.” She emphasizes that people with BPD are often coping with severe trauma, whereas diagnostic language, with its focus on personality difficulties, shifts attention and empathy away from the horror of these traumas. She claims this risks constituting a silencing and blaming perspective that pathologizes and medicalizes peoples’ coping strategies as a symptom of a psychiatric disorder located within the individual’s personality. Research of lived experiences with health and suffering, such as the present study, can have an important function in the field of mental health to allow emphatic engagement with the lived experiences of the suffering people (Natvik and Moltu, 2016), offering one contribution toward balancing epistemic injustice.

For the specific population of people diagnosed with BPD, the first person research literature on the suffering individuals’ perspectives is scarce, but not non-existent. However, a majority of the existing studies focus on how persons diagnosed with BPD experience specific intervention strategies, such as music therapy (Strehlow and Lindner, 2016), specific group therapy (Sagen Inderhaug and Karterud, 2015), and MBT (Lonargáin et al., 2017). The contributions of such studies are evident when it comes to refining intervention protocols and practices, but one critique might be that the focus on the intervention risks silencing the full service user perspective on recovery. For instance, synthesizing seven studies in an otherwise interesting and well-written meta-synthesis of how individuals diagnosed with BPD perceive DBT, Little et al. (2017) conclude that the synthesis highlights how key factors in DBT impact day-to-day life and patients’ identity, and that outcome measures may not capture the complexity and magnitude of this impact. It should be noted in this respect, that meta- and mega-analyses of close to 70 years of quantitative psychotherapy research attribute the largest effect-sizes, by far, to client factors and extra-therapeutic factors (Lambert and Ogles, 2004), illustrating our point that when researching experiences of specific interventions qualitatively, the intervention itself might occupy too much of the foreground.

Interesting examples of first-person studies include studies of the experiences of imprisoned people diagnosed with BPD, people diagnosed with BPD as one of many problems, adolescents diagnosed with BPD, and of specific phenomena, such as the experience of self, identity, and crises. Lovell and Hardy (2014) reported an IPA study of interviews with eight women with a diagnosis of BPD in a forensic setting. Their resulting themes of identity, power, protection and confusion seem to suggest that the prison context allows for different expressions of such vulnerability than do voluntary treatment contexts. Indeed, if safety and autonomy are core aspects of recovery from personality disorder problems in general (Shepherd et al., 2016), and for people diagnosed with BPD, as suggested in our findings, a forensic setting seems challenging. Spodenkiewicz et al. (2013) interviewed 50 youths diagnosed with BPD in a lifeworld study, and reported that fear-based negative feelings and experiences of social isolation or hostility were descriptive of their experiences. They also find that a sense of continuity and time is hard to hold onto. We understand these findings to add meaning to the overarching theme in the present study that recovery is about moving toward connectedness. Such a formulation holds as a premise that this process starts from a state of disconnectedness. Reporting from a thematic analysis of interviews with five women diagnosed with BPD, Agnew et al. (2016) found three identity themes, which they coin connection, distance between us, and hurt and healing. They describe how these women understood their suffering as having relational origins and relational solutions, and that in recovery, finding ways of connecting constructively to others was important. One study exploring recovery processes in a group of women diagnosed with BPD reports that feeling safe, validated, and trusted within treatment helped the women in their recovery (Holm and Severinsson, 2011). Last, in an in-depth case study of one individual’s recovery journey over 5 years, Johansen et al. (2017) describe how recovery moved via three phases, from attachment and dependence on a therapist, through working level headed together, to moving autonomously on “into the world.” These three studies, in various ways, echo the importance of relationality, agency and connection in understanding problems and recovery related to BPD.

Anthony (1993) defines personal recovery from mental health challenges as *a deeply personal, unique process of changing one’s attitudes, values, feelings, goals, skills and/or role. It is a way of living a satisfying, hopeful and contributing life even with the limitations caused by illness*, resonating with the lived experiences of the participants of getting better as an ever ongoing matter. In a medical meta-model (Wampold and Imel, 2015), the term recovery often connotes *recovery from* something, referring to removing expressed symptoms of a disorder, such as self-harm and suicidal behavior in the context of BPD. A humanistic epistemology would rather use the term in a way that connotes *recovery in*, putting the actively living person, trying to cope with all aspects of life, amongst other things burdens of illness, in the foreground (Ramon et al., 2007). This seems to be a difference that makes a difference. The latter understanding and epistemology allow for thinking of a patient as an actively living and meaning-making agent, holding intentionality and capacity to act within his or her own life. Important work in the field of mental health has established such an understanding of agency as pivotal in working toward better service (Davidson and Shahar, 2007).

Empirical work studying different illness experiences underscore agency and autonomy as healing phenomena (Moltu et al., 2012, 2017; Bjornestad et al., 2017; Hansen et al., 2017). Moltu et al. (2017) studied what good outcomes from a patient perspective, and reported that “doing differently” rather than “feeling differently” held importance to participants. As such, functioning in social roles and personal relationship take prominence over symptoms when people develop autonomy and agency. Similarly, reporting on self-agency development for people with psychosis, Bjornestad et al. (2017) underscore how environmental support and gentle pressure supports such processes. Most psychotherapy models have agency and autonomy building as central to their theories of change, and recently Lind et al. (2018) reported that people with BPD developed self-agency beyond the control group through a 12-month therapy program. Importantly in our findings, the theme “Learning to hold one’s own” underscores how, for our participants, developing agency was both about support and learning coping skills for everyday challenges. An epistemology seeing people as actively creating their own lives provides a sound foundation to build theory about health interventions part in those lives.

### Limitations of the Study

All participants in the present study were women. Although this reflects an important statistical point, 75% given the diagnosis of BPD are women (Skodol and Bender, 2003), there is clearly also a need for more knowledge on the lived experiences of men with a diagnosis of BPD. Furthermore, the present study was conducted within a Norwegian context, which means that despite that participants had experience with different treatment modalities such as mentalization-based therapy or dialectical behavioral treatment, the mental health care were all provided within the same public health system at approximately the same period. Although this exploratory study provided us with rich data, the study builds on a relatively small and homogenous group of participants. When interpreting the results the context of a uniform single payer free of charge health care system should be integrated.

Another limitation is that we conducted all interviews within the context of an ongoing therapy, which necessarily gives direction to the interviews and our analysis. As the overarching theme of “moving toward connectedness” also will involve processes outside of treatment and care, there is a need for studies focusing more explicitly on these processes as well as with various stakeholders, such as family and friends, who can provide different perspectives on these processes. Given the importance of power issues that we have discussed in this article, future studies might also consider choosing methodological approaches that are more sophisticated in capturing the complexity of such issues, for example discourse analysis.

### Implications

Though these findings needs to be carefully considered in light of the study limitations, we recommend continued training and educational efforts toward mental health professionals and staff in order to develop more humanistic approaches that increasingly recognize the trauma people with BPD has survived. Following Davidson et al. (2016), we may say that we need to “stop asking patients (implicitly) the question: “What is wrong with you?” and start asking them explicitly instead: “What has happened to you?” to be followed by the question: “And how can I be of most help?” (p. 47). Moreover, findings in this study suggest that professionals in treatment and care can be more attentive to how power issues can be present in the way we use language, describe and diagnose people, and that this perspective could benefit clinical education.

## Conclusion

Recovery for people with severe mental illnesses is a gradual and continuous process. Treatment programs can help or hinder various aspects of this process. The present study emphasize how moving toward connectedness seems to be a key dimension in the recovery process for people with BPD, comprising variations over the subthemes of “Learning to hold one’s own,” “Needing honesty and genuine mutuality,” “Daring to belong,” and “Making a room for recovery” for the one who suffers.

## Author Contributions

BK carried out the data collection and took a leading role in data analyses. EN, MV, and CM took part in the study design and analytic phases of the project, and contributed to writing the article.

## Conflict of Interest Statement

The authors declare that the research was conducted in the absence of any commercial or financial relationships that could be construed as a potential conflict of interest.

## References

[B1] Agnew G. Shannon C. Ryan T. Storey L. McDonnell C. (2016). Self and identity in women with symptoms of borderline personality: a qualitative study. *Int. J. Qual. Stud. Health Well Being* 11:30490. 10.3402/qhw.v11.30490 27015876 PMC4808078

[B2] Allen J. G. Fonagy P. (2006). *The Handbook of Mentalization-Based Treatment.* Hoboken, NJ: John Wiley & Sons 10.1002/9780470712986

[B3] American Psychiatric Association [APA] (2000). *Diagnostic and Statistical Manual of Mental Disorders-IV-TR.* Washington, DC: American Psychiatric Association.

[B4] Anthony W. A. (1993). Recovery from mental illness: the guiding vision of the mental health service system in the 1990s. *Psychosoc. Rehabil. J.* 16 11–23. 10.1037/h0095240

[B5] Bateman A. Fogany P. (1999). Effectiveness of partial hospitalization in the treatment of borderline personality disorder: a randomized controlled trial. *Am. J. Psychiatry* 156 1563–1569. 10.1176/ajp.156.10.1563 10518167

[B6] Bateman A. Fonagy P. (2008). 8-year follow-up of patients treated for borderline personality disorder: mentalization-based treatment versus treatment as usual. *Am. J. Psychiatry* 165 631–638. 10.1176/appi.ajp.2007.07040636 18347003

[B7] Bateman A. Fonagy P. (2009). Randomized controlled trial of outpatient mentalization-based treatment versus structured clinical management for borderline personality disorder. *Am. J. Psychiatry* 166 1355–1364. 10.1176/appi.ajp.2009.09040539 19833787

[B8] Bateman A. Fonagy P. (2013). Mentalization-based treatment. *Psychoanal. Inqu.* 33 595–613. 10.1080/07351690.2013.835170 26157198 PMC4467231

[B9] Bateman A. W. Fonagy P. (2003). The development of an attachment-based treatment program for borderline personality disorder. *Bull. Menninger Clin.* 67:187. 10.1521/bumc.67.3.187.23439 14621062

[B10] Bentall R. (2006). Madness explained: why we must reject the Kraepelinian paradigm and replace it with a ‘complaint-orientated’approach to understanding mental illness. *Med. Hypotheses* 66 220–233. 10.1016/j.mehy.2005.09.026 16300903

[B11] Binder P. E. Holgersen H. Moltu C. (2012). Staying close and reflexive: an explorative and reflexive approach to qualitative research on psychotherapy. *Nord. Psychol.* 64 103–117. 10.1080/19012276.2012.726815

[B12] Bjornestad J. Bronnick K. Davidson L. Hegelstad W. T. V. Joa I. Kandal O. (2017). The central role of self-agency in clinical recovery from first episode psychosis. *Psychosis* 9 140–148. 10.1080/17522439.2016.1198828

[B13] Black G. Murray J. Thornicroft G. (2014). Understanding the phenomenology of borderline personality disorder from the patient’s perspective. *J. Ment. Health* 23 78–82. 10.3109/09638237.2013.869570 24689663

[B14] Bradshaw W. Armour M. P. Roseborough D. (2007). Finding a place in the world: the experience of recovery from severe mental illness. *Qual. Soc. Work* 6 27–47. 10.1177/1473325007074164

[B15] Braun V. Clarke V. (2006). Using thematic analysis in psychology. *Qual. Res. Psychol.* 3 77–101. 10.1191/1478088706qp063oa

[B16] Davidson L. Carr E. Bellamy C. Tondora J. Fossey E. Styron T. (2016). “Principles for recovery-oriented inpatient care,” in *Handbook of Recovery in Inpatient Psychiatry*, eds Singh N. Barber J. W. Van Sant S. (Berlin: Springer), 39–58. 10.1007/978-3-319-40537-7_2

[B17] Davidson L. O’Connell M. J. Tondora J. Lawless M. Evans A. C. (2005). Recovery in serious mental illness: A new wine or just a new bottle? *Prof. Psychol. Res. Pract.* 36 480–487. 10.1037/0735-7028.36.5.480

[B18] Davidson L. Shahar G. (2007). From deficit to desire: a philosophical reconsideration of action models of psychopathology. *Philos. Psychiatry Psychol.* 14 215–232. 10.1353/ppp.0.0127

[B19] Drake R. E. Whitley R. (2014). Recovery and severe mental illness: description and analysis. *Can. J. Psychiatry* 59 236–242. 10.1177/070674371405900502 25007276 PMC4079142

[B20] Flanagan E. H. Davidson L. Strauss J. S. (2007). Issues for DSM-V: incorporating patients’ subjective experiences. *Am. J. Psychiatry* 164 391–392. 10.1176/ajp.2007.164.3.391 17329461

[B21] Flanagan E. H. Davidson L. Strauss J. S. (2010). The need for patient-subjective data in the DSM and the ICD. *Psychiatry* 73 297–307. 10.1521/psyc.2010.73.4.297 21198379

[B22] Fonagy P. Allison E. (2014). The role of mentalizing and epistemic trust in the therapeutic relationship. *Psychotherapy* 51 372–380. 10.1037/a0036505 24773092

[B23] Gullestad F. S. Wilberg T. (2011). Change in reflective functioning during psychotherapy—A single-case study. *Psychother. Res.* 21 97–111. 10.1080/10503307.2010.525759 21331977

[B24] Hansen H. Stige S. Davidson L. Moltu C. Veseth M. (2017). How do people experience early intervention services for psychosis? A meta-synthesis. *Qual. Health Res.* 28 259–272. 10.1177/1049732317735080 29039239

[B25] Hill C. E. (2012). *Consensual Qualitative Research. A Practical Resource for Investigating Social Science Phenomena.* Washington, DC: American Psychological Association.

[B26] Holm A. L. Severinsson E. (2011). Struggling to recover by changing suicidal behaviour: narratives from women with borderline personality disorder. *Int. J. Ment. Health Nurs.* 20 165–173. 10.1111/j.1447-0349.2010.00713.x 21492357

[B27] Horn N. Johnstone L. Brooke S. (2007). Some service user perspectives on the diagnosis of borderline personality disorder. *J. Ment. Health* 16 255–269. 10.1111/papt.12156 29034599

[B28] Husserl E. (1954/1970). *The Crisis of European Sciences and Trancendental Phenomenology. An Introduction to Phenomenological Philosophy.* Evanston, IL: Northwestern University Press.

[B29] Johansen A. B. Tavakoli S. Bjelland I. Lumley M. (2017). Constructivist simultaneous treatment of borderline personality disorder, Trauma, and addiction comorbidity: a qualitative case study. *Qual. Health Res.* 27 236–248. 10.1177/1049732315618659 26701963

[B30] Kellogg S. H. Young J. E. (2006). Schema therapy for borderline personality disorder. *J. Clin. Psychol.* 62 445–458. 10.1002/jclp.20240 16470629

[B31] Kliem S. Kröger C. Kosfelder J. (2010). Dialectical behavior therapy for borderline personality disorder: a meta-analysis using mixed-effects modeling. *J. Consult. Clin. Psychol* 78 936–951. 10.1037/a0021015 21114345

[B32] Knox S. Burkard A. W. (2009). Qualitative research interviews. *Psychother. Res.* 19 566–575. 10.1080/10503300802702105 19579087

[B33] Kvale S. Brinkmann S. (2009). *InterViews: Learning the Craft of Qualitative Research Interviewing.* London: Sage.

[B34] Kyratsous M. Sanati A. (2017). Epistemic injustice and responsibility in borderline personality disorder. *J. Eval. Clin. Pract.* 23 974–980. 10.1111/jep.12609 27491561

[B35] Lam D. C. Salkovskis P. M. Hogg L. I. (2016). ‘Judging a book by its cover’: an experimental study of the negative impact of a diagnosis of borderline personality disorder on clinicians’ judgements of uncomplicated panic disorder. *Br. J. Clin. Psychol.* 55 253–268. 10.1111/bjc.12093 26212734

[B36] Lambert M. J. Ogles B. M. (2004). “The efficacy and effectiveness of psychotherapy,” in *Handbook of Psychotherapy and Behavior Change*, 5th Edn, ed. Lambert M. J. (New York, NY: Wiley).

[B37] Lavik K. O. Veseth M. Frøysa H. Binder P. E. Moltu C. (2018). What are “good outcomes” for adolescents in public mental health settings? *Int. J. Ment. Health Syst.* 12:3. 10.1186/s13033-018-0183-5 29387146 PMC5775535

[B38] Leamy M. Bird V. Le Boutillier C. Williams J. Slade M. (2011). Conceptual framework for personal recovery in mental health: systematic review and narrative synthesis. *Br. J. Psychiatry* 199 445–452. 10.1192/bjp.bp.110.083733 22130746

[B39] Lieb K. Zanarini M. C. Schmahl C. Linehan M. M. Bohus M. (2004). Borderline personality disorder. *Lancet* 364 453–461. 10.1016/S0140-6736(04)16770-6 15288745

[B40] Lind M. Jørgensen C. R. Heinskou T. Simonsen S. Bøye R. Thomsen D. K. (2018). Patients with borderline personality disorder show increased agency in life stories after 12 months of psychotherapy. *Psychotherapy* 10.1037/pst0000184 [Epub ahead of print]. 30113186

[B41] Linehan M. (1993). *Cognitive-Behavioral Treatment of Borderline Personality Disorder.* New York, NY: The Guilford Press.

[B42] Linehan M. M. Cochran B. N. Kehrer C. (2001). “Dialectical behavior therapy for borderline personality disorder,” in *Clinical Handbook of Psychological Disorders: A Step-By-Step Treatment Manual*, ed. Barlow D. H. (New York, NY: The Guilford Press), 365–420.

[B43] Linehan M. M. Comtois K. A. Murray A. M. Brown M. Z. Gallop R. J. Heard H. L. (2006). Two-year randomized controlled trial and follow-up of dialectical behavior therapy vs therapy by experts for suicidal behaviors and borderline personality disorder. *Arch. Gen. Psychiatry* 63 757–766. 10.1001/archpsyc.63.7.757 16818865

[B44] Linehan M. M. Schmidt H. Dimeff L. A. Craft J. C. Kanter J. Comtois K. A. (1999). Dialectical behavior therapy for patients with borderline personality disorder and drug-dependence. *Am. J. Addict.* 8 279–292. 10.1080/105504999305686 10598211

[B45] Lingiardi V. McWilliams N. (2015). The psychodynamic diagnostic manual–2nd edition (PDM-2). *World Psychiatry* 14 237–239. 10.1002/wps.20233 26043343 PMC4471982

[B46] Lingiardi V. McWilliams N. (2017). *Psychodynamic Diagnostic Manual: PDM-2.* New York, NY: Guilford Publications.

[B47] Little H. Tickle A. das Nair R. (2017). Process and impact of dialectical behaviour therapy: a systematic review of perceptions of clients with a diagnosis of borderline personality disorder. *Psychol. Psychother.* 91 278–301. 10.1111/papt.12156 29034599

[B48] Lonargáin D. Ó Hodge S. Line R. (2017). Service user experiences of mentalisation-based treatment for borderline personality disorder. *Ment. Health Rev. J.* 22 16–27. 10.1108/MHRJ-04-2016-0008

[B49] Lovell L. J. Hardy G. (2014). Having a diagnosis of borderline personality disorder in a forensic setting: a qualitative exploration. *J. Forensic Pract.* 16 228–240. 10.1108/JFP-01-2014-0003

[B50] Miller S. G. (1994). Borderline personality disorder from the patient’perspective. *Hosp. Community Psychiatry* 45 1215–1219. 7868105 10.1176/ps.45.12.1215

[B51] Moltu C. Binder P. E. Stige B. (2012). Collaborating with the client in the struggle toward growth: skilled psychotherapists’ experiences of the patient in difficult therapies ending well. *J. Psychother. Integr.* 22 85–108. 10.1037/a0028010

[B52] Moltu C. Stefansen J. Nøtnes J. C. Skjølberg Å Veseth M. (2017). What are «good outcomes» in public mental health settings? A qualitative exploration of clients’ and therapists’ experiences. *Int. J. Ment. Health Syst.* 11:12. 10.1186/s13033-017-0119-5 28101132 PMC5237476

[B53] Natvik E. Moltu C. (2016). Just experiences? Ethical contributions of phenomenologically-oriented research. *Scand. Psychol.* 3:e17 10.15714/scandpsychol.3.e17

[B54] Nehls N. (1999). Borderline personality disorder: the voice of patients. *Res. Nurs. Health* 22 285–293. 10.1002/(SICI)1098-240X(199908)22:4<285::AID-NUR3>3.0.CO;2-R 10435546

[B55] Olfson M. Blanco C. Wall M. Liu S.-M. Saha T. D. Pickering R. P. (2017). National trends in suicide attempts among adults in the United States. *JAMA Psychiatry* 74 1095–1103. 10.1001/jamapsychiatry.2017.2582 28903161 PMC5710225

[B56] Pilgrim D. (2013). The failure of diagnostic psychiatry and some prospects of scientific progress offered by critical realism. *J. Crit. Real.* 12 336–358. 10.1179/1476743013Z.0000000004

[B57] Ramon S. Healy B. Renouf N. (2007). Recovery from mental illness as an emergent concept and practice in Australia and the UK. *Int. J. Soc. Psychiatry* 53 108–122. 10.1177/0020764006075018 17472085

[B58] Sagen Inderhaug T. Karterud S. (2015). A qualitative study of a mentalization-based group for borderline patients. *Group Anal.* 48 150–163. 10.1177/0533316415577341

[B59] Shepherd A. Sanders C. Doyle M. Shaw J. (2016). Personal recovery in personality disorder: systematic review and meta-synthesis of qualitative methods studies. *Int. J. Soc. Psychiatry* 62 41–50. 10.1177/0020764015589133 26081467

[B60] Skodol A. E. Bender D. S. (2003). Why are women diagnosed borderline more than men? *Psychiatr. Q.* 74 349–360. 14686459 10.1023/a:1026087410516

[B61] Spodenkiewicz M. Speranza M. Taïeb O. Pham-Scottez A. Corcos M. Révah-Levy A. (2013). Living from day to day–qualitative study on borderline personality disorder in adolescence. *J. Can. Acad. Child Adolesc. Psychiatry* 22 282–289. 24223047 PMC3825468

[B62] Stoffers J. M. Lieb K. (2015). Pharmacotherapy for borderline personality disorder—current evidence and recent trends. *Curr. Psychiatry Rep.* 17:534. 10.1007/s11920-014-0534-0 25413640

[B63] Stone M. H. (1989). *The Course of Borderline Personality Disorder.* Washington, DC: American Psychiatric Press.

[B64] Strehlow G. Lindner R. (2016). Music therapy interaction patterns in relation to borderline personality disorder (BPD) patients. *Nord. J. Music Ther.* 25 134–158. 10.1080/08098131.2015.1011207

[B65] Sulzer S. H. (2015). Does “difficult patient” status contribute to de facto demedicalization? The case of borderline personality disorder. *Soc. Sci. Med.* 142 82–89. 10.1016/j.socscimed.2015.08.008 26298091 PMC4870819

[B66] Swartz M. Blazer D. George L. Winfield I. (1990). Estimating the prevalence of borderline personality disorder in the community. *J. Pers. Disord.* 4 257–272. 10.1521/pedi.1990.4.3.257

[B67] Torgersen S. Kringlen E. Cramer V. (2001). THe prevalence of personality disorders in a community sample. *Arch. Gen. Psychiatry* 58 590–596. 10.1001/archpsyc.58.6.590 11386989

[B68] Veseth M. Binder P. E. Borg M. Davidson L. (2012). Toward caring for oneself in a life of intense ups and downs: a reflexive-collaborative exploration of recovery in bipolar disorder. *Qual. Health Res.* 22 119–133. 10.1177/1049732311411487 21653886

[B69] Veseth M. Binder P.-E. Stige S. H. (2017). “If there’s no stability around them”: experienced therapists’ view on the role of patients’ social world in recovery in bipolar disorder. *Int. J. Ment. Health syst.* 11:55. 10.1186/s13033-017-0166-y 28943889 PMC5607588

[B70] Wampold B. Imel Z. E. (2015). *The Great Psychotherapy Debate. The Evidence for What Makes Psychoterapy Work.* 2nd Edn. New York, NY: Routledge 10.4324/9780203582015

[B71] Ware N. C. Hopper K. Tugenberg T. Dickey B. Fisher D. (2007). Connectedness and citizenship: redefining social integration. *Psychiatr. Serv.* 58 469–474. 10.1176/ps.2007.58.4.469 17412847

[B72] Watts J. (2017). *Testimonial Injustice and Borderline Personality Disorder.* Available at: https://www.huffingtonpost.co.uk/dr-jay-watts/testimonial-injustice-and_b_14738494.html?guccounter=1 [Accessed 18, May 2018].

[B73] Young S. L. Ensing D. S. (1999). Exploring recovery from the perspective of people with psychiatric disabilities. *Psychiatr. Rehabil. J.* 22 219–231. 10.1037/h0095240

